# Circadian Disruption Accelerates Tumor Growth and Angio/Stromagenesis through a Wnt Signaling Pathway

**DOI:** 10.1371/journal.pone.0015330

**Published:** 2010-12-23

**Authors:** Yoshihiro Yasuniwa, Hiroto Izumi, Ke-Yong Wang, Shohei Shimajiri, Yasuyuki Sasaguri, Kazuaki Kawai, Hiroshi Kasai, Takashi Shimada, Koichi Miyake, Eiji Kashiwagi, Gen Hirano, Akihiko Kidani, Masaki Akiyama, Bin Han, Ying Wu, Ichiro Ieiri, Shun Higuchi, Kimitoshi Kohno

**Affiliations:** 1 Department of Molecular Biology, School of Medicine, University of Occupational and Environmental Health, Kitakyushu, Japan; 2 Department of Clinical Pharmacokinetics, Graduate School of Pharmaceutical Sciences, Kyushu University, Fukuoka, Japan; 3 Department of Pathology and Cell Biology, School of Medicine, University of Occupational and Environmental Health, Kitakyushu, Japan; 4 Department of Environmental Oncology, Institute of Industrial Ecological Science, University of Occupational and Environmental Health, Kitakyushu, Japan; 5 Department of Biochemistry and Molecular Biology, Nihon Medical School, Tokyo, Japan; 6 Department of Urology, Shengjing Hospital, China Medical University, Shenyang, China; 7 Department of Oncology, First Affiliated Hospital, China Medical University, Shenyang, China; National Institute of Diabetes and Digestive and Kidney Diseases/National Institutes of Health, United States of America

## Abstract

Epidemiologic studies show a high incidence of cancer in shift workers, suggesting a possible relationship between circadian rhythms and tumorigenesis. However, the precise molecular mechanism played by circadian rhythms in tumor progression is not known. To identify the possible mechanisms underlying tumor progression related to circadian rhythms, we set up nude mouse xenograft models. HeLa cells were injected in nude mice and nude mice were moved to two different cases, one case is exposed to a 24-hour light cycle (L/L), the other is a more “normal” 12-hour light/dark cycle (L/D). We found a significant increase in tumor volume in the L/L group compared with the L/D group. In addition, tumor microvessels and stroma were strongly increased in L/L mice. Although there was a hypervascularization in L/L tumors, there was no associated increase in the production of vascular endothelial cell growth factor (VEGF). DNA microarray analysis showed enhanced expression of WNT10A, and our subsequent study revealed that WNT10A stimulates the growth of both microvascular endothelial cells and fibroblasts in tumors from light-stressed mice, along with marked increases in angio/stromagenesis. Only the tumor stroma stained positive for WNT10A and WNT10A is also highly expressed in keloid dermal fibroblasts but not in normal dermal fibroblasts indicated that WNT10A may be a novel angio/stromagenic growth factor. These findings suggest that circadian disruption induces the progression of malignant tumors via a Wnt signaling pathway.

## Introduction

Modern lifestyles and the use of indoor lighting mean that many people are exposed to a long photoperiod throughout the year [1]. This is most evident in shift workers, especially night workers. This results in the disruption of circadian rhythms, which is known to induce many different types of stress [2]. Abnormal circadian rhythms, including exposure to light at night, are associated with a higher cancer risk and a poorer prognosis [3]–[7], which may be one of the reasons that the incidence of cancer is increasing in individuals subjected to these stresses. Circadian genes have been shown to function as oncogenes or tumor suppressors at both the systemic and cellular levels due to their roles in cell proliferation, cell cycle regulation, apoptosis and DNA damage signaling pathways [8], [9]. However, the molecular or systemic mechanisms involved in tumor growth under artificial illumination stress conditions have not been identified. In fact, the question of whether artificial illumination stress promotes tumor growth at all is still controversial [10], [11]. To identify the possible mechanisms underlying tumor progression related to circadian rhythms, we set up nude mouse xenograft models and revealed that artificial light stress induced tumor growth and angio/stromagenesis through WNT10A overexpression.

## Results

### Circadian disruption induces tumor growth and angio/stromagenesis

**Figure 1:**
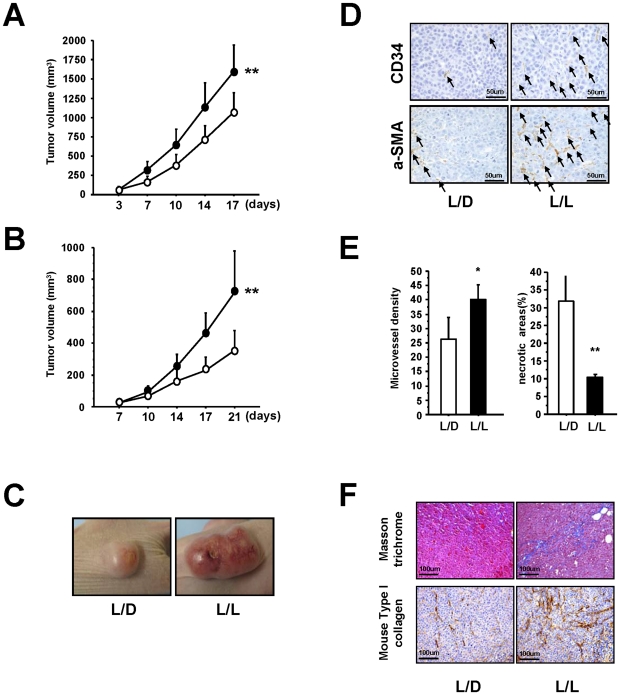
Effect of photoperiod manipulation on the growth of human HeLa cell or PC3 cell tumors. (A) Hela cell tumors. Volume of the subcutaneous xenografts in nude mice housed in either L/L (closed circle; *n* = 16) or L/D (open circle; *n* = 16) conditions. After 17 days of growth, L/L tumors were significantly larger than L/D tumors (*F* from repeated measure ANOVA = 12.276, **P<0.01). (B) PC3 cell tumors. Volume of the subcutaneous xenografts in nude mice housed in either L/L (*n* = 8) or L/D (*n* = 8) conditions. A significant reduction in volume is seen in the L/D tumors compared with the L/L tumors (*F* from repeated measure ANOVA  = 18.360, **P<0.01). (C) Representative photograph of L/L and L/D tumors showing the obvious difference in size. (D) Immunohistochemical analysis of CD34 positive (CD34^+^) cells and aSMA positive (aSMA^+^) cells in the L/L and L/D tumors. Increased numbers of CD34^+^ and aSMA^+^ (black arrows) are clearly visible in the L/L tumors. (E) The number of microvessels was quantified using the number of CD34^+^ cells. An increase in microvessel density (vessels in 10 viewing fields; *n* = 3 per group, *P<0.05), is accompanied by a decrease in necrosis (*n* = 3 per group **P<0.01). (F) Representative photographs showing masson trichrome staining of the expanded extracellular matrix and immunohistochemical analysis of mouse Type I collagen in the L/D and L/L tumors.

The mice were divided into two groups: one group was exposed to 24-hour periods of artificial light (L/L) the other was exposed to a more conventional 12-hour light/dark cycle (L/D). First, we examined the effect of light stress on the *in vivo* growth of epidermoid cancer (HeLa) cell tumors and found a significant increase in tumor volume in the L/L group compared with the L/D group (Figure 1A). Similar results were obtained using a xenograft model incorporating prostate cancer (PC3) cells (Figures 1B). Examples of the Hela cell tumors in the L/L and L/D groups are shown in Figure 1C and Figure S1. The L/L tumors were not only larger, but also immunohistochemical analysis showed them to be highly vascular, with increased numbers of CD34 positive (CD34^+^) and a-Smooth Muscle Actin (a-SMA) positive (a-SMA^+^) cells (Figure 1D). High vascularity of tumor surface in L/L group using HeLa cells was reproducibly observed in four independent experiments. Also, the microvessel density within the L/L tumors was significantly higher than that in the L/D tumors and correlated with a reduction in the amount of necrosis (Figure 1E). Masson trichrome staining of the tumor stroma showed a clear expansion of the extracellular matrix (ECM; stained blue) in the L/L tumors not seen in the L/D tumors (Figure 1F). The immunostaining of mouse Type I collagen also showed the increase of ECM in the L/L tumors (Figure 1F). Taken together, these results clearly show that abnormal circadian rhythms induce marked tumor growth accompanied by increased angio/stromagenesis.

### Microarray analysis of L/L tumors and L/D tumors

**Figure 2:**
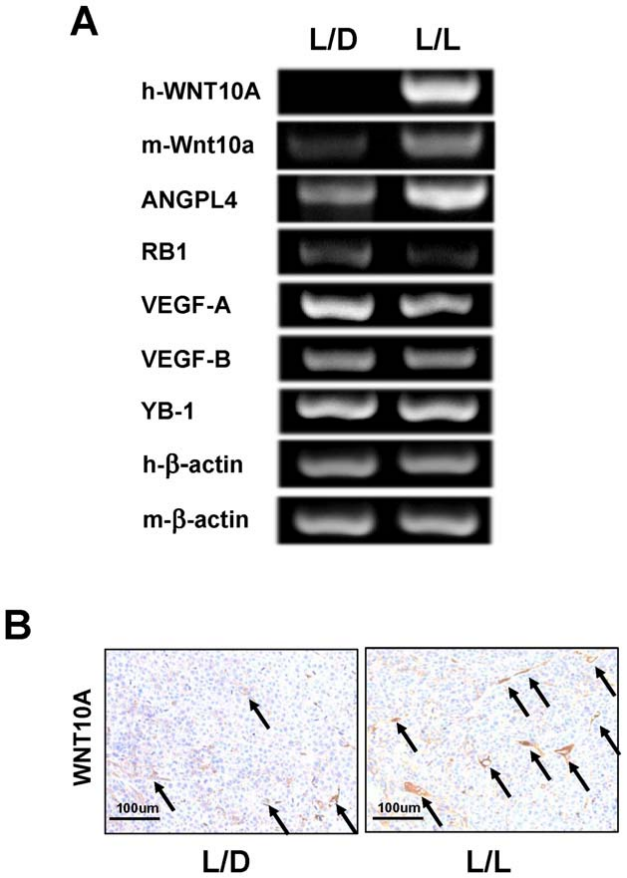
WNT10A was upregulated in L/L tumors. (A) RT-PCR of the relevant gene transcripts was carried out based on the results of the DNA microarray analysis. Human WNT10A (h-WNT10), mouse Wnt10a (m-Wnt10a) and ANGPL4 were upregulated in L/L mice tumors. RB1 was downregulated. There was no difference in the expression levels of VEGF-A, VEGF-B and YB-1 between L/L and L/D tumors. Human β-actin (h-β-actin) and mouse β-actin (m-β-actin) were used as internal control. The cycle number is 30 for all semi-quantitative RT-PCR except h-WNT10A. Nested PCR technique to investigate the expression of human WNT10A in tumors. The cycle number of 1st PCR is 30 and that of 2nd nested PCR is 35. (B) Immunohistochemical analysis of WNT10A in L/L and L/D mice tumors. The arrows indicate tumor blood vessels.

Next, we wanted to investigate the molecular mechanisms underlying the striking morphological differences between L/L and L/D tumors. Whole-genome expression DNA microarray analysis was performed to identify the genes and biological pathways that might be regulated by photoperiod manipulation. We found that 201 genes were transcriptionally upregulated in the L/L tumors compared with the L/D tumors (Table S1). Surprisingly, the expression of human VEGF-A and VEGF-B, which are the most important molecules in cancer angiogenesis, was the same in L/L and L/D tumors (Table S1 and Figure 2A), suggesting that a novel angiogenic factor is involved in increased L/L tumor growth. We focused on genes encoding secretory proteins (Table 1) and found a greater than 9-fold upregulation in the expression of WNT10A in L/L tumors compared with L/D tumors. We designed human WNT10A and mouse Wnt10a specific primers for semi-quantitative RT-PCR analysis and checked the specificity of the primers (Figures S2A and Table S2). Semi-quantitative RT-PCR showed that not only human WNT10A, but also mouse Wnt10a, was upregulated in L/L tumors (Figure 2A); however, the expression of human WNT10A in the L/L tumors was still very low as it could only be detected using nested techniques (1st PCR 30 cycles and 2nd PCR 35 cycles). Immunohistochemical analysis showed WNT10A expression mainly around the blood vessels and it was increased in L/L tumors compared with L/D tumors (arrows in Figure 2B), indicating that this enhanced expression of WNT10A is derived from mouse tissues.

**Table 1:** The subset of secretory protein genes was selected if fold change marked more than 2.0 in the L/L tumor sample compared to the L/D tumor sample.

Gene symbol	Accession number	Fold change	Gene discription
WNT10A	NM_025216	9.41	Protein WNT-10A precursor.
CCL11	NM_002986	3.06	Eotaxin precursor (Small inducible cytokine A11).
MUC1	NM_002456	2.79	Mucin-1 precursor.
SCGB1C1	NM_145651	2.51	Secretoglobin family 1C member 1 precursor.
ANGPTL4	NM_016109	2.36	Angiopoietin-related protein 4 precursor (Angiopoietin-like 4).
CES1	NM_001266	2.30	Liver carboxylesterase 1 precursor.
EDN1	NM_001955	2.20	Endothelin-1 precursor.
AMY2A	NM_000699	2.19	Pancreatic alpha-amylase precursor.
C1QTNF6	NM_031910	2.13	Complement C1q tumor necrosis factor-related protein 6 precursor.
HTRA3	NM_053044	2.08	Probable serine protease HTRA3 precursor.
FBLN1	NM_006486	2.04	Fibulin-1 precursor.
APOC1	NM_001645	2.03	Apolipoprotein C-I precursor.

### WNT10A overexpression cells induce tumor growth, angiogenesis and stromagenesis in vivo xenograft models

**Figure 3:**
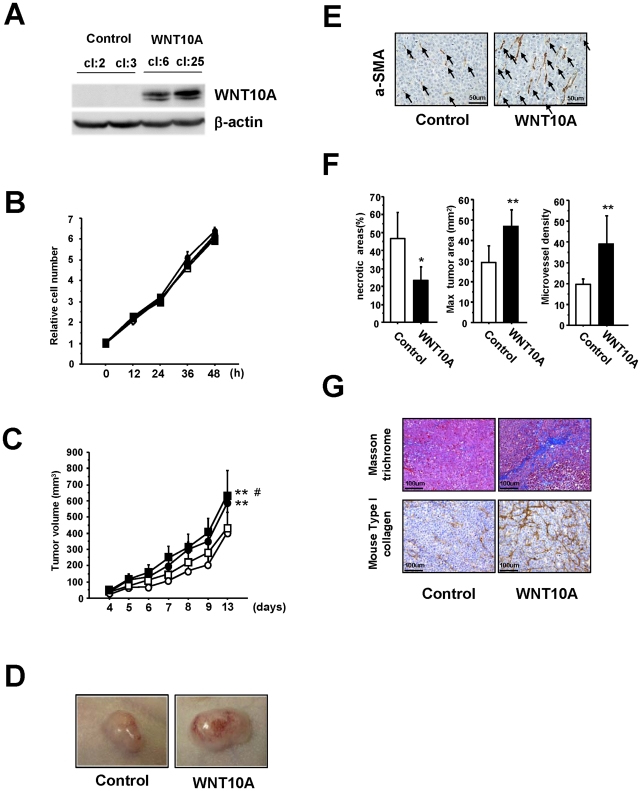
WNT10A functions as an angio/stromagenetic growth factor *in vivo* xenograft models. (A) Establishment of a stable WNT10A-overexpressing cell line. (B), (C) The growth rate of these stable cell lines (B) *in vitro* and (C) *in vivo*. Two control cell lines (cl:2; open circle, cl:3; open square) and two stable WNT10A-overexpressing cell lines (cl:6; closed circle, cl:25, closed square) were used. **P<0.01 compared with the control cl:2 group and #*P*<0.05 compared with the control cl:3 group using Scheffe's test. *n* = 8 per groups. (D) Representative photograph WNT10A-overexpressing tumors in nude mice illustrating their hypervascular nature. (E) Immunostaining of tumors with an anti-aSMA antibody. Increased numbers of aSMA^+^ cells (black arrows) are clearly visible in WNT10A-overexpressing tumors. (F) Reduced areas of tissue necrosis in WNT10A-overexpressing tumors are accompanied by increased tumor size and increased microvessel density (*n* = 4 or 6 per group, *P<0.05 and **P<0.01). Microvessel density was quantified using the number of aSMA^+^ cells (G) Masson trichrome staining showing expansion of the extracellular matrix and immunohistchemical analysis of mouse Type I collagen in the control and WNT10A-overexpressing tumors.

To further investigate the role played by WNT10A in these morphological changes, we established another nude mouse model implanted with HeLa cells overexpressing WNT10A (Figures 3A and S3). Because the growth rate of these WNT10A-overexpressing cells was similar to that of control cells *in vitro* (Figure 3B), we were surprised to see that the growth rate of the implanted WNT10A-overexpressing tumors was faster than that of control tumors (Figure 3C). Furthermore, as shown in Figure 3D, most of the tumors were hypervascular; even those from mice housed under L/D conditions. Immunohistochemical analysis of these WNT10A-overexpressing tumors showed increased numbers of a-SMA^+^ cells coupled with increased size, microvessel density, significantly reduced areas of necrosis (Figures 3E and 3F) and an expanded ECM (Figure 3G).

### WNT10A is expressed in fibroblasts and WNT10A stimulates the growth of both fibroblasts and vascular endothelial cells in vitro

**Figure 4:**
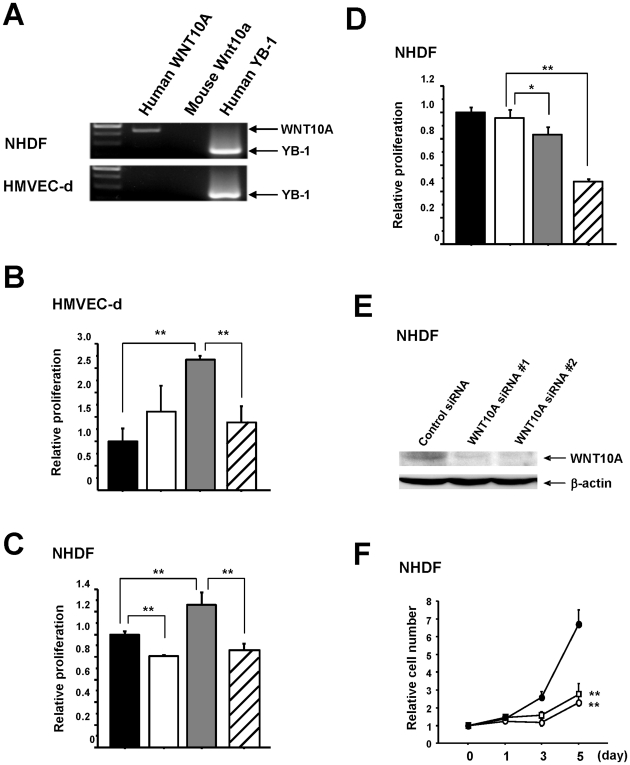
WNT10A is expressed in fibroblast cells and functions as an angio/stromagenesis growth factor *in vitro*. (A) RT-PCR for human WNT10A mRNA in NHDF and HMVEC-d cells. Human YB-1 was used as a positive control and mouse Wnt10a was used as a negative control. (B), (C) WNT10A-dependent growth of HMVEC-d and NHDF cells. For the proliferation assays using BrdU incorporation, HMVEC-d (NHDF) cells were cultured in conditioned medium (CM) with or without WNT10A antibody for 24 hours (black bar; Control-CM, white bar; Control-CM + WNT10A antibody 5 µg/ml, gray bar; WNT10A-CM, slash bar; WNT10A-CM + WNT10A antibody 5 µg/ml). **P<0.01. *n* = 3 per groups. (D) WNT10A-dependent autocrine growth of NHDF cells. NHDF cells were cultured with the recommended medium (RM) with or without WNT10A antibody (black bar; RM, white bar; RM + IgG 5 µg/ml, gray bar; RM + WNT10A antibody 2 µg/ml, slash bar; RM + WNT10A antibody 5 µg/ml). *P<0.05 and **P<0.01. *n* = 3 per groups. (E) Complete knockdown of WNT10A expression in NHDF cells is achieved using the two siRNAs against WNT10A. Whole-cell extracts (100 µg) were subjected to SDS-PAGE, and Western blotting analysis was performed using the indicated antibodies. (F) Knockdown of WNT10A suppresses the growth of NHDF cells (Control siRNA; closed circle, WNT10A siRNA #1; open square, WNT10A siRNA #2; open circle). **P<0.01 compared with the control siRNA group.

Based on these results, we hypothesized that WNT10A was functioning as a growth factor for both vascular endothelial cells and fibroblasts and was involved in a novel mechanism of tumor growth, possibly via the promotion of angio/stromagenesis. To confirm this hypothesis, we used RT-PCR to show that normal human dermal fibroblasts (NHDF) cells express WNT10A, but normal human dermal microvascular endothelial (HMVEC-d) cells do not (Figure 4A). This suggests the presence of a WNT10A-dependent autocrine growth system in fibroblasts. Cell proliferation analysis showed that the growth of both HMVEC-d and NHDF cells was stimulated by the addition of conditioned medium from WNT10A-overexpressing cells and was significantly inhibited by the addition of an anti-WNT10 antibody (Figures 4B and 4C). NHDF cells cultured in recommended medium were also effectively inhibited by the addition of the anti-WNT10A antibody (Figure 4D). In addition, knockdown of WNT10A-expression using siRNA inhibited the growth of NHDF cells (Figures 4E and 4F), confirming the existence of a WNT10A-dependent autocrine growth mechanism.

### Tumor stroma cells express WNT10A

**Figure 5:**
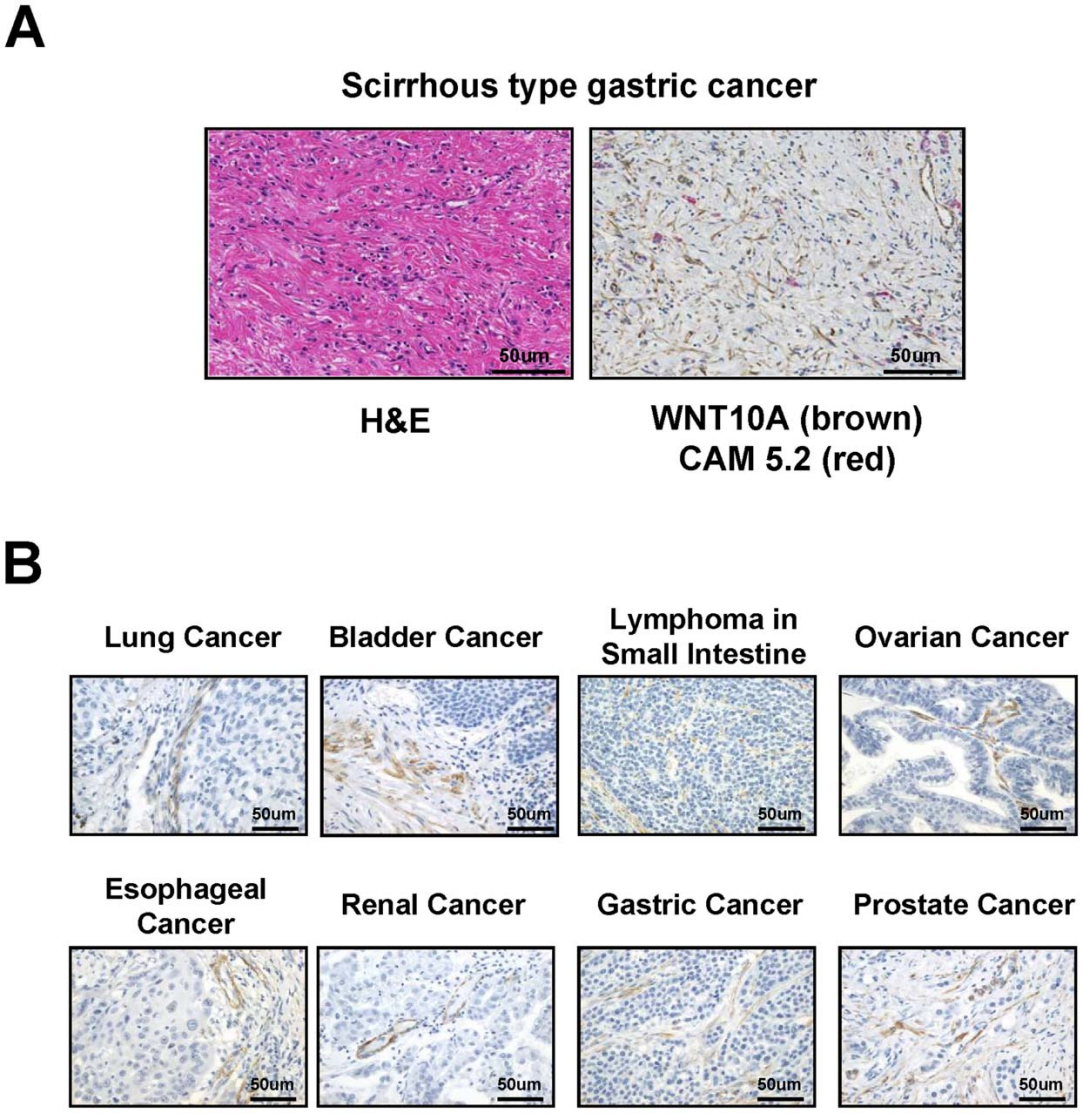
Immunohistochemical analysis of WNT10A expression in various human cancer specimens. (A) H&E and double immunohistochemical staining of scirrhous-type signet-ring carcinoma cells. 3,3′-Diaminobenzidine (DAB) was used as chromogen for WNT10A staining (brown color) and Vulcan fast red was used for cytokeratin CAM 5.2 staining (red color). Anti-cytokeratin CAM 5.2 was used for staining of signet-ring cell carcinoma cells. (B) WNT10A expresses cancer stroma cells in various human cancer specimens. 3,3′-Diaminobenzidine (DAB) was used as chromogen for WNT10A staining (brown color).

The pattern of WNT10A expression in human tumors was examined by immunohistochemistry (Figures 5A and 5B). A careful examination of the double stained tissues showed marked increase of WNT10A positive fibroblastic cells in scirrhous type gastric cancer which is a representative cancer with hyperplastic stroma (Figure 5A). Only the tumor stroma stained positive for WNT10A, strongly suggesting expression by stromal cells such as fibroblasts, myofibroblasts, smooth muscle cells, and pericytes.

### WNT10A is expressed in keloid stroma, but not in normal dermal stroma

**Figure 6:**
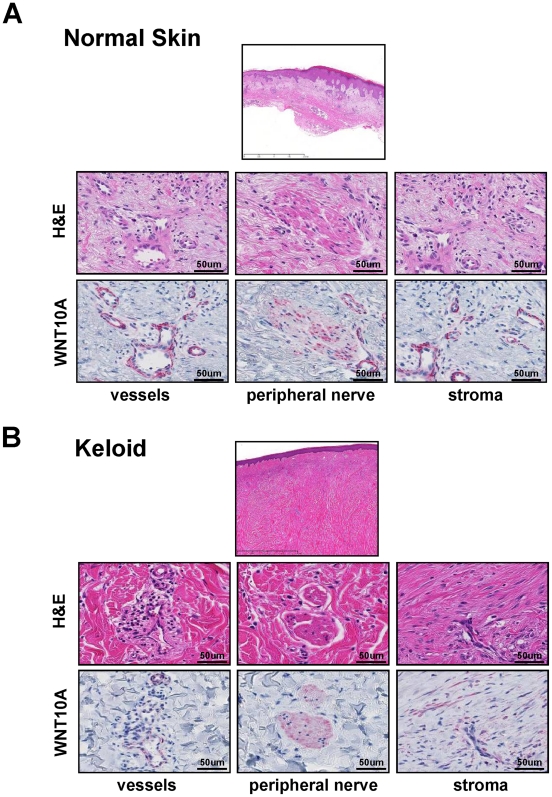
WNT10A is expressed in stromal cells of keloid tissue. H&E and anti-WNT10A antibody staining in (A) normal skin and (B) keloid tissue. WNT10A was expressed around the vessels and in the peripheral nerve cells of normal skin, but not in stromal cells. In contrast, WNT10A was expressed around vessels, in peripheral nerve cells and strongly in stromal cells of keloid tissue. Vulcan fast red was used for WNT10A staining (red color).

The inducible expression of Wnt genes, including WNT10A, stimulates the proliferation of hepatic progenitor cells [12], and mutations in WNT10A are associated with an autosomal recessive ectodermal dysplasia [13], [14]. In addition, The expression of Wnt signaling antagonists has been shown to be down-regulated in keloid, which is an aggressive wound healing tissue, fibroblasts [15], [16]. These previous reports indicate that WNT signaling is involved in both tissue repair and wound healing. Because an old hypothesis suggests that cancer results from uncontrolled wound-healing [17], we investigated WNT10A expression in keloid tissue. WNT10A-positive cells were only found in the vessels and peripheral nerves of normal skin (Figure 6A). On the other hand, WNT10A expression markedly increased in fibroblastic cells in the hyperplastic stroma of keloid tissue (Figure 6B), suggesting that WNT10A functions as an angio/stromagenesis gene in tumor progression, thus supporting the “old” hypothesis. Although WNT10A expression was observed in cutured normal human dermal fibroblasts (Figure 4E), WNT10A expression was not in fibroblast in normal skin (Figure 6A). This may be probably due to the sensitivity of immunostaining analysis. Another possibility is that normal human dermal fibroblasts were cultured with growth factors which may induce the WNT10A expression.

### Oxidative stress induce WNT10A expression

**Figure 7:**
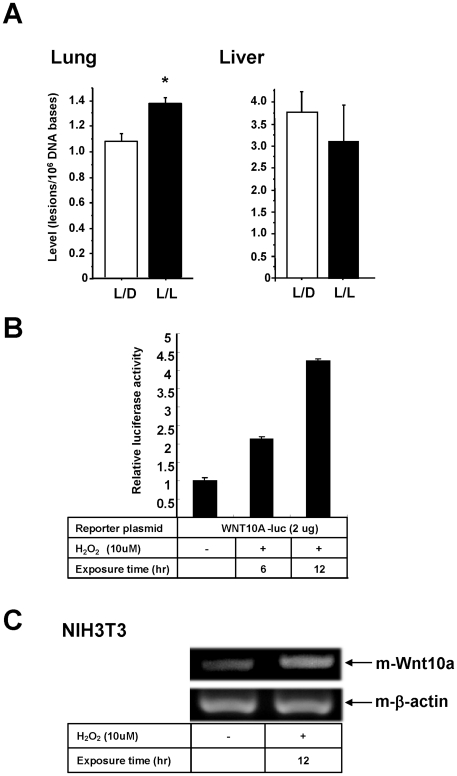
WNT10A is induced by oxidative stress. (A) 8-hydroxydeoxyguanosine levels are significantly increased in the lung tissues of L/L compared with L/D mice (*P<0.05). (B) Reporter assays. The promoter activity of the WNT10A gene was measured using a luciferase system after the addition of hydrogen peroxide. 42 hours after transfection (exposure time 6 hr) or 36 hours after transfection (exposure time 12 hr) of the reporter plasmid into PC3 cells, cells were treated with 10 µM of hydrogen peroxide. Luciferase activities were assayed after 48 hours of transfection. The results shown are normalized against protein concentrations measured using the Bradford method and are representative of at least three independent experiments. (C) Induction of mouse Wnt10a transcripts by oxidative stress. NIH3T3 cells were treated with or without H_2_O_2_ (10 µM) for 12 hours. Total RNAs were assayed by semi- quantitative RT-PCR. Mouse β-actin was used for internal control.

The level of psychological and physiological stress experienced by the mice is hard to measure experimentally. So, we measured 8-OH deoxyguanosine (8-OH-dG) associated with increased levels of oxidative stress. We found that the level of 8-OH-dG in lung tissue, but not liver, from L/L mice was significantly higher than that in L/D mice (Figure 7A). This is consistent with the fact that lung tissue is more sensitive to oxidative DNA damage than other tissues [18]. This data strongly suggest an association between disruption of circadian rhythms and increased oxidative stress responses. A preliminary study also showed that the promoter activity of the WNT10A gene was induced by the oxidizing agent, hydrogen peroxide and Wnt10a mRNA transcript level was also increased in NIH3T3 cells treated with hydrogen peroxide (Figures 7B and 7C). These provide further evidence supporting the role of oxidative stress in tumor promotion and progression.

## Discussion

Greater understanding of the complexity of the tumor microenvironment, and the role of tumor angiogenesis, will lead to further advances in cancer treatment [4], [19], [20]. The results presented in this paper are both interesting and unexpected and strongly suggest that disruption of circadian rhythms promotes tumor growth through WNT10A-dependent angio/stromagenesis resulting from increased levels of oxidative stress. The transcriptional factor of NF-κB is activated by oxidative stress or tumor necrosis factor alpha (TNF-α) [21]. WNT10A has been shown to be one of the NF-κB target genes and it's expression was induced by TNF-α [22], [23]. Since there is one NF-kB site in the promoter region of WNT10A gene, it is conceivable that WNT10A might be regulated by NF-κB pathway. WNT signaling pathway has been implicated in angiogenesis [24] and tumor stroma microenvironment [25]. These data suggest that both endothelial cells and stromal cells are activated by WNT signals from cancer cells. On the other hand, our data indicate that both endothelial cells and stromal cells may be activated by WNT10A signals from non tumor cells, such as cancer associated fibroblasts. WNT signaling has been separated into a “canonical” pathway or “non-canonical” pathways [26]. Since canonical WNT signaling pathway stabilize β-catenin, we hypothesized that WNT10A might also stabilize β-catenin. The expression of β-catenin was observed in the endothelial cells of newly formed tumor vessels (Figure S4), suggesting that Wnt/β-catenin signaling plays a role in tumor angiogenesis. WNT signaling is also known to play an important role in cancer and stem cell biology [27], indicating that WNT10A might affect not only the tumor microenvironment, but also stem cells themselves.

There are some limitations in this study. We cannot exclude the possibility that other physiological and/or hormonal factors, such as melatonin, affected the growth of the implanted cancer cells in our mouse models [28]–[30]. Subcutaneous injection of rapidly growing human cancer cells into nude mice provided a setting in which tumor growth could be assessed in a relatively short time span. Orthotopic model will be a better way to confirm our results because it more accurately reproduces the interactions between tumor cells and their microenvironment [31]. Nevertheless, our data clearly show that WNT10A has angio/stromagenic activity. Further analysis is required to clarify whether WNT10A-Frizzled binding mediates cell proliferation in both endothelial cells and stromal cells. Examining WNT10A receptors and associated signal transduction pathways may provide valuable insights into the role of circadian rhythms in tumor progression [32], [33]. Our findings not only support the emerging links between circadian rhythm, oxidative stress and tumor progression at the molecular level, but also warn of the adverse effects of artificial light.

## Materials and Methods

### Primary cells, cell lines and culture conditions

HMVEC-d and NHDF cells were purchased from Lonza Co. HMVEC-d and NHDF cells were maintained with EGM-2-MV BulletKit and FGM-2 BulletKit (Lonza Co), respectively. HMVEC-d cells were cultured in endothelial cell basic medium (EBM) containing 5% FBS and a growth factor mixture containing hydrocortisone, ascorbic acid, FGF, VEGF, IGF, EGF and gentamycin. NHDF cells were cultured in fibroblast basic medium (FBM) containing 2%, FBS containing the appropriate growth factors (insulin, FGF, and gentamycin). The human prostate cancer cell line PC3 was kindly gifted by Dr M Nakagawa (Kagoshima University, Kagoshima, Japan) [34]. Although HeLa cell line was kindly gifted by Dr S Akiyama (Tokushima University, Tokushima, Japan) as human epidermoid cancer KB cell line [35], we carried out STR profiling at National Institute of Biomedical Innovation in Japan and revealed that KB cell line is same as Hela cell line. Mouse fibroblast NIH3T3 cell line was obtained from the Japanese Cancer Research Resources Bank (JCRB) [36]. HeLa cells and human prostate cancer PC3 cells were cultured in Eagle's minimal essential medium as described previously [37], [38]. NIH3T3 cells were cultured in Dulbecco's modified Eagle's minimal essential medium. These mediums were purchased from Nissui Seiyaku (Tokyo, Japan) and contained 10% fetal bovine serum. Cell lines were maintained in a 5% CO_2_ atmosphere at 37°C.

### Anti-WNT10A antibody

A polyclonal antibody was raised against WNT10A by multiple immunization of a New Zealand white rabbit with synthetic peptides. The synthetic peptide sequences were RKLHRLQLDALQRGKGLSHGVPEHPALPC (aa 172–199) and CGGQLEPGPAGAPSPAPGAPGPRRRASPA (aa 307–334). This antibody was used for the Western blot and cell proliferation assays. For the cell proliferation assays, antibodies were purified from both control and WNT10A antisera using protein G columns (Mab Trap, Amersham Pharmacia Biotech).

### Mouse studies

All protocols were approved by the Ethics Committee of Animal Care and Experimentation, University of Occupational and Environmental Health (admission number; AE-07039), and were performed according to the Institutional Guidelines for Animal Experiments and to Law (number 105) and Notification (number 6) of the Japanese government. All surgery was performed under anesthetization (mixture of ketamine 50 mg/kg and medetomidine 1 mg/kg), and all efforts were made to minimize suffering. Eight-week-old male nude mice (BALB/c nu/nu; Kyudo Co.) were used for subcutaneous xenografting. Mice were injected with 100 µl (1×10^6^ cells) of Hela cells or PC3 cells suspension at two separate dorsal sites. The subcutaneous xenegrafting experiments were carried out four times for HeLa cells and twice for PC3 cells. Mice were randomly caged (5/cage) and subdivided into L/L and L/D groups. Tumor volume was measured using the two principal perpendicular diameters: V =  length (mm) × (width (mm))^2^ ×1/2.

### Preparation of human tissue samples

Human normal skin, keloid tissue and cancer samples from different organ were examined in the Department of Pathology and Cell Biology at University of Occupational and Environmental Health in Kitakyushu, Japan. The diagnosis was re-evaluated and confirmed by at least three board-certified surgical pathologists who had examined formalin-fixed, paraffin-embedded tissue sections stained with haematoxylin and eosin (H&E) or other appropriate immunohistochemical stains.

### Immunohistochemistry and histpathology

Formalin-fixed tumors (transplanted to mice or human cancer specimens), normal human dermal tissues and human keloid tissues were embedded in paraffin and sections were immunostained using anti-CD34 (1∶50; Immunotech), anti-aSMA (1∶150, DAKO), anti-mouse Type I collagen (1∶250; AbD Serotec), anti-WNT10A (1∶50, Sigma-Aldrich) and anti-cytokeratin CAM5.2 (1∶10, Becton Dickinson) according to the manufacturer's instructions. The anti-WNT10A antibody recognizes both human and mouse WNT10A. 3,3′-Diaminobenzidine (DAB) or Vulcan fast red were used as chromogen. The necrotic area/tumor area ratio was evaluated using NanoZoomer Digital Pathology Virtual Slide Viewer software (Hamamatsu Photonic Co.). Masson trichrome staining is used for evaluating extracellular matrix. All procedures were approved by the ethics committee of the University of Occupational and Environmental Health.

### DNA microarray analysis and RT-PCR

DNA microarray analysis was performed using 3-DGene (Toray Industries). All data is MIAME compliant and that raw data has been deposited in a MIAME compliant database (GSE23969). Only one tumor from each L/D and L/L group which represent the typical look of tumors size and color was used for RNA preparation in same experiment. Total RNA was isolated from tumors and cultured cells using QIAshredder and RNeasy-Mini kits (Qiagen). RT-PCR was performed using the Qiagen OneStep RT-PCR kit. The primers used in this study are listed in Table S2. Cycle number is 30 excluding some exceptions. The cycle number of these exceptions is listed in each figure legend. Human specificity of h-WNT10A primers is shown using NHDF cells (Figure 4A) and mouse specificity of m-Wnt10a primers is shown using NIH3T3 cells (Figure S2A). Specificity of human and/or mouse β-actin primers is shown using Hela cells and NIH3T3 cells (Figure S2B).

### Plasmid construction

WNT10A cDNA was constructed by PCR using a superscript cDNA library (Invitrogen) (Table S2). The PCR product was cloned into the pGEM-T easy vector (Promega) and the full-length cDNA fragment was recloned into the pcDNA3.1 vector (Invitrogen). To prepare the reporter plasmid containing the promoter region of the human WNT10A gene, PCR of human genomic DNA was performed using the appropriate primers (listed in Table S2). The PCR product was then cloned into the pGL3-basic vector (Promega).

### Cloning of stable transfectants

HeLa cells were transfected with pcDNA3.1-WNT10A using the Effectene reagent (Qiagen) and cultured with 500 µg/ml hygromycin for 15–20 days. The resulting colonies were isolated and the cellular expression levels of WNT10A in each clone analyzed by Western blotting with an anti-WNT10A antibody.

### Western blotting analysis

Whole-cell lysates were prepared as previously described [38], [39]. The 100 µg of whole-cell lysates were separated by sodium dodecyl sulfate-polyacrylamide gel electrophoresis (SDS-PAGE) and transferred to polyvinylidene difluoride (PVDF) microporous membranes (Millipore, Billerica, MA, USA) using a semi-dry blotter. The blotted membranes were treated with 5% (w/v) skimmed milk in 10 mM Tris, 150 mM NaCl and 0.2% (v/v) Tween 20, and incubated for 1 h at room temperature with the primary antibody. The following antibodies and dilutions were used: 1∶1,000 dilution of anti-WNT10A and 1∶5,000 dilution of anti-β-actin. The membranes were then incubated for 45 min at room temperature with a peroxidase-conjugated secondary antibody, visualized using an ECL kit (GE Healthcare Bio-Science, Buckinghamshire, England, UK). The detection was performed with LAS-4000 mini (FUJIFILM).

### WNT10A siRNA knockdown

Twenty-five base-pair double-stranded RNA oligonucleotides were commercially generated (Invitrogen) (Table S3). siRNA transfections were performed according to the manufacturer's instructions with modifications (Invitrogen) [38], [40]. Stealth™ RNAi Negative control Duplexes (Cat. No. 12935-300; Invitrogen) was used as control siRNA. Whole cell extracts (100 µg) were prepared from fibroblasts 72 hrs after siRNA transfection and Western blotting was performed.

### Conditioned Media (CM)

Stable transfectants were cultured in MEM containing 10% FBS until they formed confluent monolayers. The MEM was then replaced with either conditioned EBM (all growth factors and FBS are 0.1 fold compared to normal EBM) or conditioned FBM (insulin, FGF and FBS are 0.1 fold compared to normal FBM) for 24 hours, after which the medium was collected. The CM was then centrifuged and filtered to remove cells and debris. Control-CM was prepared from the culture medium of growing control-cl2 cells, and WNT10A-CM was prepared from the cultured medium of growing WNT10A-cl25 cells.

### Cell Proliferation Assays

WNT10A-overexpressing cell lines and control cell lines were seeded in 12-well plates and counted every 12 hours. NHDF cells were seeded in 12-well plates and transfected with siRNA as described above. For the purposes of analysis, “0 hours” was taken to be 12 hours post transfection. The cells were harvested with trypsin and counted with a Coulter-type cell size analyzer (CDA-500, Sysmex). BrdU was incorporated using a cell proliferation ELISA kit (Roche Diagnostics).

### Luciferase assay

Transient transfection and luciferase assays were performed as previously described [40]. Briefly, PC3 cells (1×10^5^) were seeded into 12-well plates and, one day later, transfected with the WNT10A reporter plasmid using the Superfect reagent (Qiagen). Finally, the cells were incubated under normal culture conditions, or in the presence of 10 µmol/L (10 µM) H_2_O_2_. Forty-eight hours post-transfection, the cells were lysed with reporter lysis buffer (Promega) and luciferase activity was detected using a Picagene kit (Toyoinki). The results shown are normalized against protein concentrations measured using the Bradford method and are representative of at least three independent experiments.

### Measurement of 8-hydroxydeoxyguanosine

The amount of 8-hydroxydeoxyguanosine (8-OH-dG) present in the cellular DNA was measured using a high performance liquid chromatography (HPLC)-electrochemical detector (ECD) system as previously described [41]. The final 8-OH-dG value was calculated as the number of 8-OH-dG residues/10^6^ dG residues.

### Statistical analysis

We compared continuous variables with repeated measure analysis of variance (ANOVA), and differences between groups were determined by Scheffe's test. Student *t* test was used for statistical analysis of the variables between the two groups. All error bars indicate standard deviation.

## Supporting Information

Figure S1 **Comparison of HeLa cell tumors in L/L and L/D mice.** Eight representative tumors are shown.
(TIF)

Figure S2 **Analysis of specificity of mouse Wnt10a primers, human β-actin primers and mouse β-actin primers.** (A) Mouse Wnt10a primers amplified mouse Wnt10a transcripts derived from mouse fibroblast NIH3T3 cells, but human WNT10A primers did not. (B) Human β-actin primers amplified human β-actin transcripts derived from human Hela cells, but mouse β-actin did not. Mouse β-actin primers amplified mouse β-actin transcripts derived from mouse fibroblast NIH3T3 cells, but human β-actin primers did not. The cycle number is 40 for all RT-PCR.
(TIF)

Figure S3 **Immunohistochemical analysis of WNT10A in control tumors and WNT10A-overexpressing tumors.**
(TIF)

Figure S4 **Immunohistochemical analysis of β-catenin in L/D and L/L tumors, and control tumors and WNT10A-overexpressing tumors.**
(TIF)

Table S1 **Genes differentially expressed between L/L tumor and L/D tumor samples. The list of selected genes with fold change marked >2.0 between L/L tumor and L/D tumor samples.**
(DOC)

Table S2 **Primers used for construction of reporter plasmid, protein expression plasmid and semi-quantitative RT-PCR.**
(DOC)

Table S3 **Double-stranded RNA 25-base pair oligonucleotides used for WNT10A kockdown analysis.**
(DOC)
