# Supplementary figures and images for: Circadian Disruption Accelerates Tumor Growth and Angio/Stromagenesis through a Wnt Signaling Pathway
Source: PLoS One. 2010 Dec 23;5(12):e15330. doi: 10.1371/journal.pone.0015330 (PMC3009728; doi:10.1371/journal.pone.0015330)

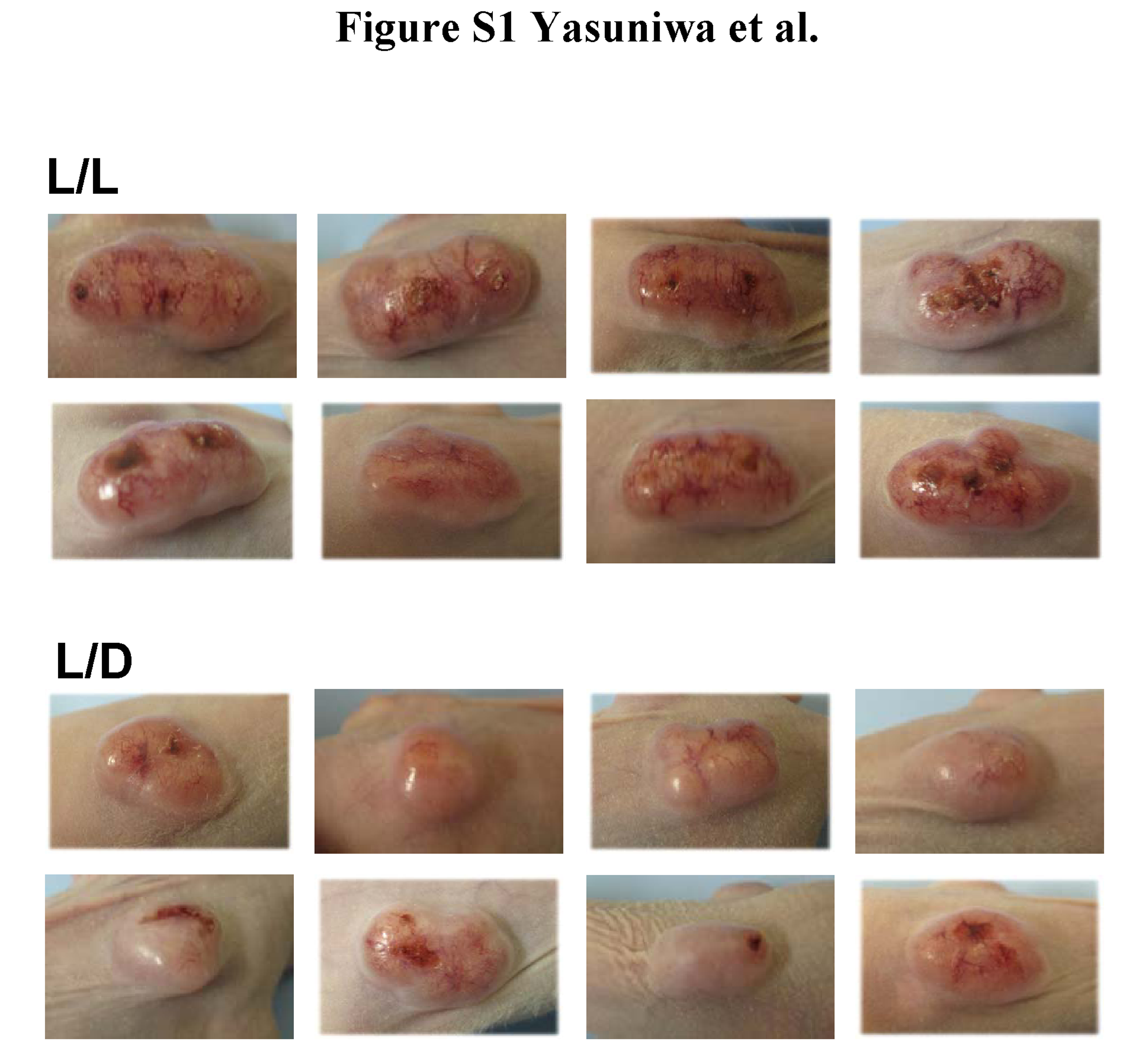

Supplement: Figure S1 — Comparison of HeLa cell tumors in L/L and L/D mice. Eight representative tumors are shown. (TIF) [file pone.0015330.s001.tif]

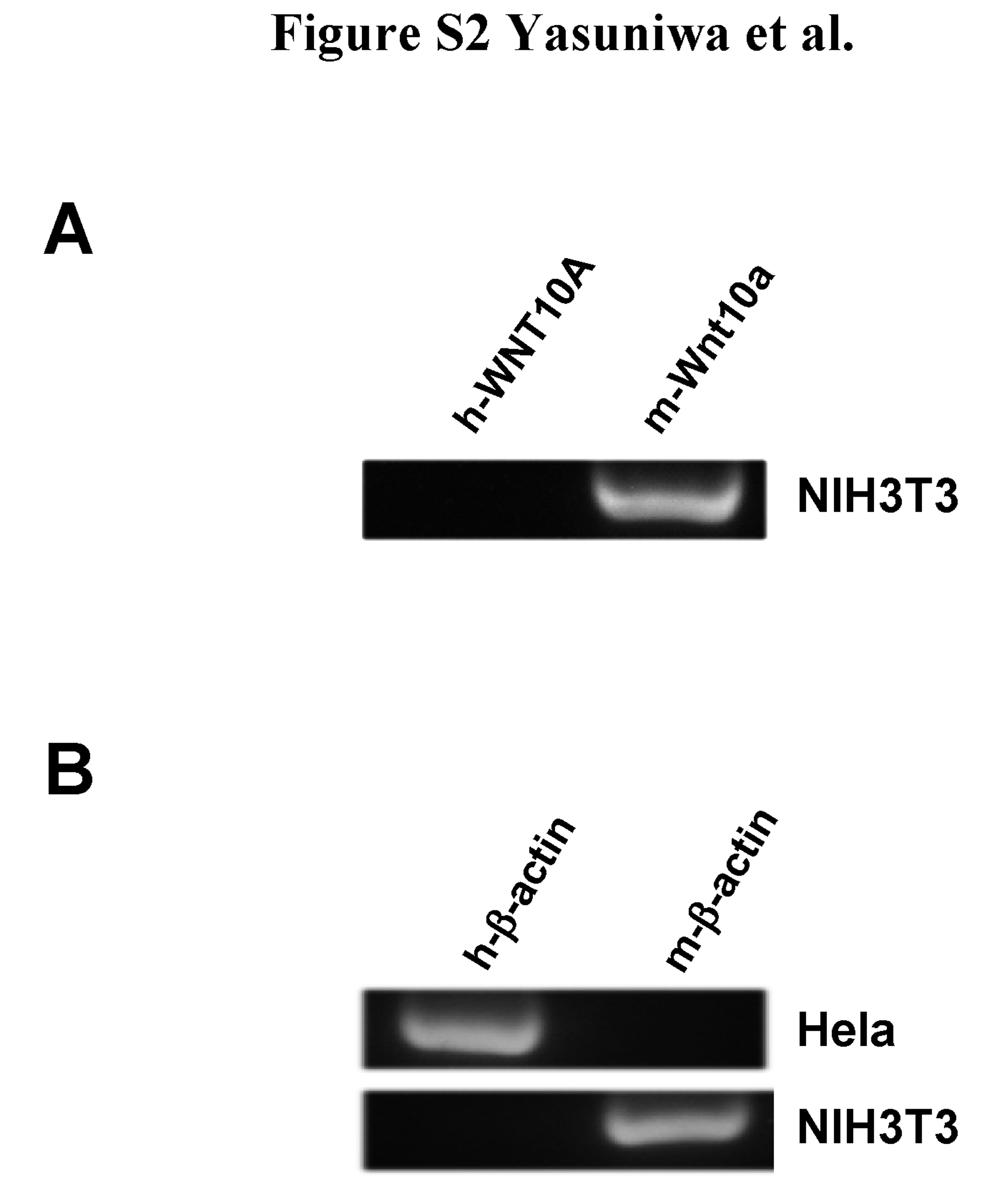

Supplement: Figure S2 — Analysis of specificity of mouse Wnt10a primers, human β-actin primers and mouse β-actin primers. (A) Mouse Wnt10a primers amplified mouse Wnt10a transcripts derived from mouse fibroblast NIH3T3 cells, but human WNT10A primers did not. (B) Human β-actin primers amplified human β-actin transcripts derived from human Hela cells, but mouse β-actin did not. Mouse β-actin primers amplified mouse β-actin transcripts derived from mouse fibroblast NIH3T3 cells, but human β-actin primers did not. The cycle number is 40 for all RT-PCR. (TIF) [file pone.0015330.s002.tif]

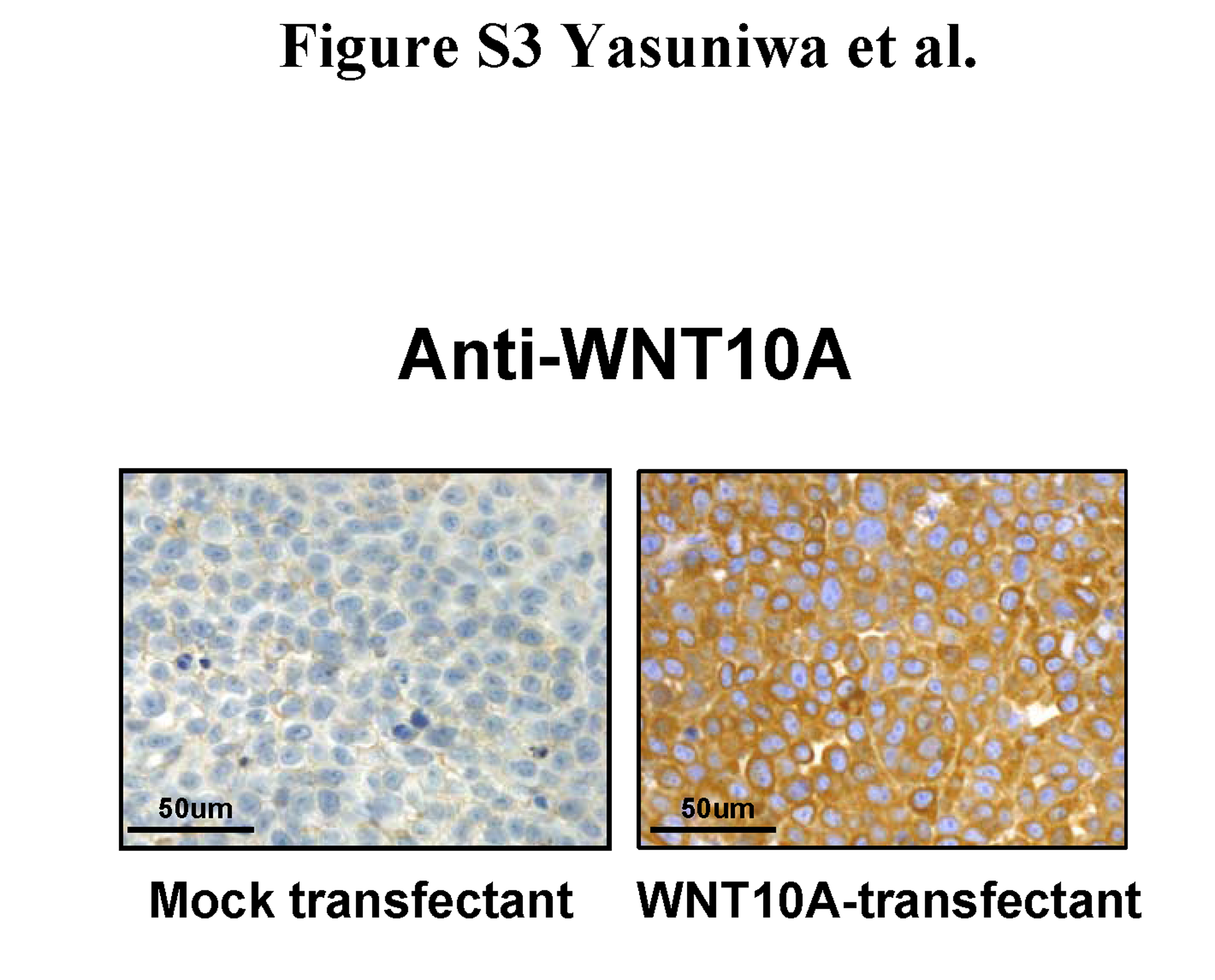

Supplement: Figure S3 — Immunohistochemical analysis of WNT10A in control tumors and WNT10A-overexpressing tumors. (TIF) [file pone.0015330.s003.tif]

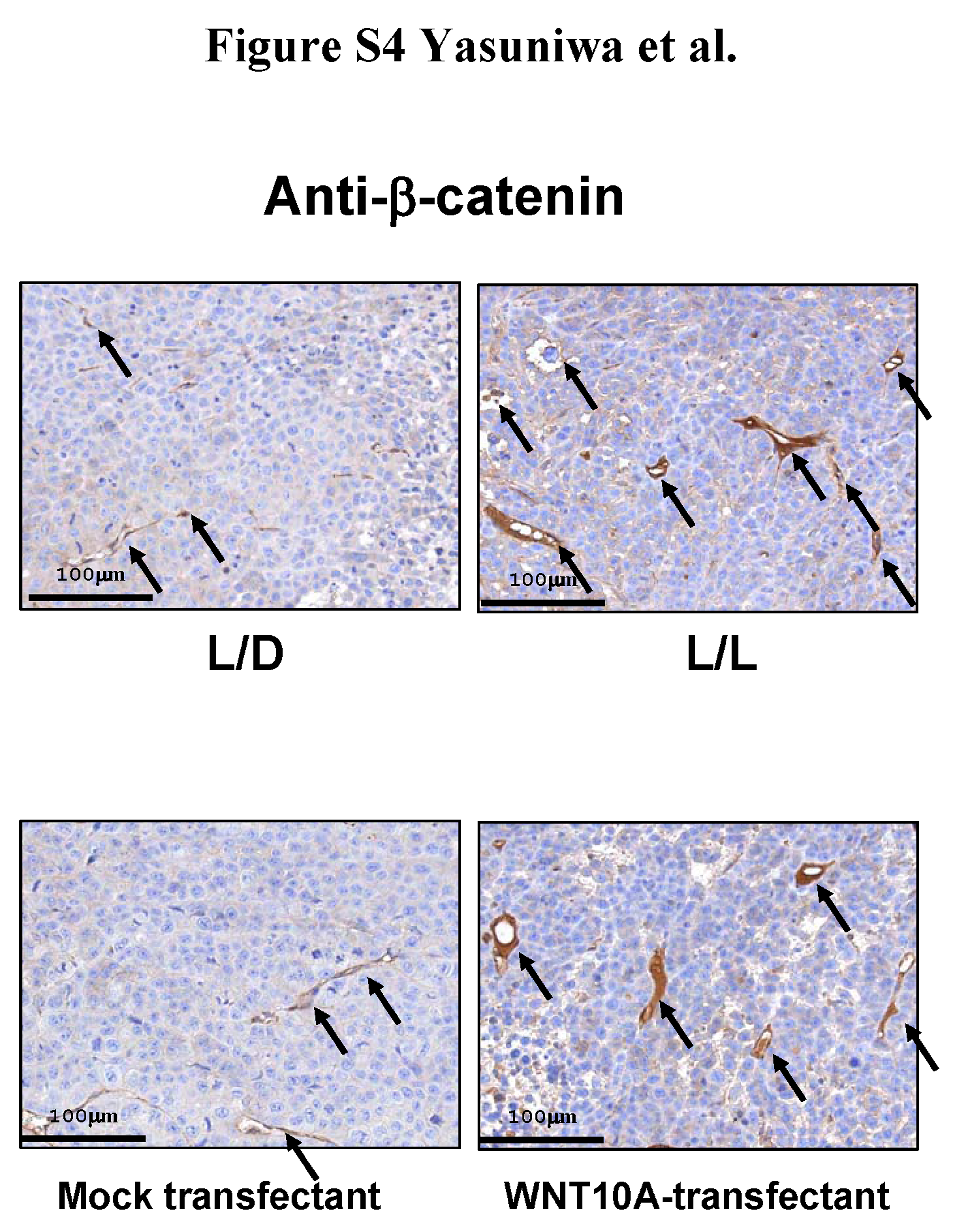

Supplement: Figure S4 — Immunohistochemical analysis of β-catenin in L/D and L/L tumors, and control tumors and WNT10A-overexpressing tumors. (TIF) [file pone.0015330.s004.tif]
