# Supplementary material for: Circadian Disruption Accelerates Tumor Growth and Angio/Stromagenesis through a Wnt Signaling Pathway
Source: PLoS One. 2010 Dec 23;5(12):e15330. doi: 10.1371/journal.pone.0015330 (PMC3009728; doi:10.1371/journal.pone.0015330)
Supplement: Table S1 — Genes differentially expressed between L/L tumor and L/D tumor samples. The list of selected genes with fold change marked >2.0 between L/L tumor and L/D tumor samples. (DOC) [file pone.0015330.s005.doc]

| Table S1. Genes differentially expressed between L/L tumor and L/D tumor samples. The list of selected genes with fold change marked >2.0 between L/L tumor and L/D tumor samples. | | | |
| --- | --- | --- | --- |
|  |  |  |  |
| Gene symbol | Accession number | Fold change | Gene discription |
|
| WNT10A | NM_025216 | 9.41 | Protein WNT-10A precursor. |
| OR5M9 | NM_001004743 | 4.49 | Olfactory receptor 5M9. |
| CSMD3 | NM_198123 | 4.01 | CUB and sushi domain-containing protein 3 precursor. |
| JMJD1C | NM_032776 | 3.88 | jumonji domain containing 1C. |
| SLC44A3 | NM_152369 | 3.44 | Choline transporter-like protein 3. |
| METT10D | NM_024086 | 3.43 | methyltransferase 10 domain containing. |
| ELMO1 | NM_014800 | 3.37 | Engulfment and cell motility protein 1. |
| SMARCC1 | NM_003074 | 3.36 | SWI/SNF-related matrix-associated actin-dependent regulator of chromatin subfamily C member 1. |
| WWP2 | NM_007014 | 3.14 | NEDD4-like E3 ubiquitin-protein ligase WWP2. |
| C3orf24 | NM_173472 | 3.13 | Uncharacterized protein C3orf24. |
| C4orf36 | NM_144645 | 3.09 | Uncharacterized protein C4orf36. |
| TOB2 | NM_016272 | 3.09 | Transducer of erbB-2 2. |
| CCL11 | NM_002986 | 3.06 | Eotaxin precursor (Small inducible cytokine A11). |
| ICAM1 | NM_000201 | 2.98 | Intercellular adhesion molecule 1 precursor. |
| Q6P5A4_HUMAN | NM_005541 | 2.90 | INPP5D protein (Fragment). |
| TRIM68 | NM_018073 | 2.90 | Tripartite motif-containing protein 68. |
| MAP2K5 | NM_145160 | 2.89 | Dual specificity mitogen-activated protein kinase kinase 5. |
| PWWP2 | NM_138499 | 2.86 | PWWP domain-containing protein 2. |
| FCRL5 | NM_031281 | 2.85 | Fc receptor-like protein 5 precursor. |
| NP_690872.2 | NM_152908 | 2.82 | H+/organic cation antiporter. |
| Q9P1R7_HUMAN | XM_496539 | 2.80 | HDCMB45P (Fragment). |
| MUC1 | NM_002456 | 2.79 | Mucin-1 precursor. |
| C1orf83 | NM_153035 | 2.76 | Chromosome 1 open reading frame 83. |
| CCDC123 | NM_032816 | 2.68 | coiled-coil domain containing 123. |
| FAM114A1 | NM_138389 | 2.65 | family with sequence similarity 114, member A1. |
| PALM | NM_002579 | 2.65 | Paralemmin. |
| ZNF121 | NM_001008727 | 2.64 | Zinc finger protein 121. |
| NP_078926.2 | NM_024650 | 2.64 | CDNA: FLJ22531 fis, clone HRC12890. |
| CCDC107 | NM_174923 | 2.62 | coiled-coil domain containing 107. |
| RHD | NM_016124 | 2.61 | Blood group Rh(D) polypeptide (Rhesus D antigen). |
| NSD1 | NM_172349 | 2.55 | Nuclear receptor-binding SET domain-containing protein 1. |
| NP_056993.2 | NM_015909 | 2.55 | neuroblastoma-amplified protein. |
| SCGB1C1 | NM_145651 | 2.51 | Secretoglobin family 1C member 1 precursor. |
| CEBPA | NM_004364 | 2.47 | CEBPA CCAAT/enhancer binding protein (C/EBP), alpha. |
| AIF1L | NM_031426 | 2.47 | allograft inflammatory factor 1-like. |
| SUMF2 | NM_015411 | 2.47 | Sulfatase-modifying factor 2 precursor. |
| FLOT2 | NM_004475 | 2.46 | Flotillin-2. |
| PTDSS2 | NM_030783 | 2.45 | Phosphatidylserine synthase 2. |
| RPS6KA1 | NM_002953 | 2.43 | Ribosomal protein S6 kinase alpha-1. |
| SDC4 | NM_002999 | 2.43 | Syndecan-4 precursor. |
| PFKFB4 | NM_004567 | 2.42 | 6-phosphofructo-2-kinase/fructose-2,6-biphosphatase 4. |
| PYCRL | NM_023078 | 2.41 | pyrroline-5-carboxylate reductase-like. |
| C15orf42 | NM_152259 | 2.41 | Chromosome 15 open reading frame 42. |
| SALL4 | NM_020436 | 2.40 | Sal-like protein 4. |
| SPHK2 | NM_020126 | 2.40 | Sphingosine kinase 2. |
| ACCN2 | NM_020039 | 2.40 | amiloride-sensitive cation channel 2, neuronal. |
| PBX2_HUMAN | NM_002586 | 2.40 | Pre-B-cell leukemia transcription factor 2. |
| PRKRIP1 | NM_024653 | 2.39 | PRKR interacting protein 1. |
| TNNT2 | NM_000364 | 2.39 | Troponin T, cardiac muscle. |
| SELO_HUMAN | NM_031454 | 2.39 | Selenoprotein O. |
| ADCY7 | NM_001114 | 2.38 | Adenylate cyclase type 7. |
| COASY | NM_025233 | 2.38 | Bifunctional coenzyme A synthase. |
| LIPH | NM_139248 | 2.37 | Lipase member H precursor. |
| ANGPTL4 | NM_139314 | 2.36 | Angiopoietin-related protein 4 precursor. |
| IL20RB | NM_144717 | 2.36 | Interleukin-20 receptor beta chain precursor. |
| PAPD5 | NM_022447 | 2.35 | PAP associated domain containing 5 isoform a. |
| C19orf2 | NM_003796 | 2.34 | Chromosome 19 open reading frame 2. |
| VPS37B | NM_024667 | 2.31 | vacuolar protein sorting 37B. |
| C16orf57 | NM_024598 | 2.31 | Chromosome 16 open reading frame 57. |
| ASNS | NM_001673 | 2.30 | Asparagine synthetase. |
| CES1 | NM_001266 | 2.30 | Liver carboxylesterase 1 precursor. |
| CD33 | NM_001772 | 2.30 | Myeloid cell surface antigen CD33 precursor. |
| BFSP1 | NM_001195 | 2.30 | Beaded filament structural protein 1. |
| PHPT1 | NM_014172 | 2.29 | Phosphohistidine phosphatase 1. |
| MSLN | NM_005823 | 2.29 | Mesothelin precursor. |
| UBQLN4 | NM_020131 | 2.28 | Ubiquilin-4. |
| IHPK1 | NM_153273 | 2.28 | Inositol hexaphosphate kinase 1. |
| C10orf10 | NM_007021 | 2.27 | Chromosome 10 open reading frame 10. |
| BCR | NM_004327 | 2.27 | Breakpoint cluster region protein. |
| MED18 | NM_017638 | 2.27 | mediator of RNA polymerase II transcription, subunit 18 homolog. |
| WIPF2 | NM_133264 | 2.26 | WAS/WASL interacting protein family member 2. |
| OTUD5 | NM_017602 | 2.26 | OTU domain containing 5. |
| FAM62A | NM_015292 | 2.25 | Protein FAM62A. |
| DCTN5 | NM_032486 | 2.24 | Dynactin subunit 5. |
| ABP1 | NM_001091 | 2.24 | Amiloride-binding protein 1. |
| NMT2 | NM_004808 | 2.24 | N-myristoyltransferase 2. |
| C20orf177 | NM_022106 | 2.24 | Chromosome 20 open reading frame 177. |
| DNAJB1 | NM_006145 | 2.23 | DnaJ homolog subfamily B member 1. |
| LNX2 | NM_153371 | 2.23 | Ligand of Numb-protein X 2. |
| C3orf59 | NM_178496 | 2.23 | Chromosome 3 open reading frame 59. |
| UROS | NM_000375 | 2.23 | Uroporphyrinogen-III synthase. |
| ALDOC | NM_005165 | 2.23 | aldolase C, fructose-bisphosphate. |
| PLXNA2 | NM_025179 | 2.23 | Plexin-A2 precursor. |
| CPSF1 | NM_013291 | 2.22 | Cleavage and polyadenylation specificity factor subunit 1. |
| RBM14 | NM_006328 | 2.22 | RNA-binding motif protein 14. |
| RFXANK | NM_003721 | 2.21 | Regulatory factor X-associated ankyrin-containing protein. |
| PMM2 | NM_000303 | 2.21 | Phosphomannomutase 2. |
| ANXA11 | NM_001157 | 2.21 | Annexin A11. |
| LAPTM5 | NM_006762 | 2.21 | Lysosomal-associated transmembrane protein 5. |
| NP_060401.2 | NM_017931 | 2.21 | CDNA FLJ20699 fis, clone KAIA2372. |
| EHMT1 | NM_024757 | 2.20 | Euchromatic histone-lysine N-methyltransferase 1. |
| MAN2B1 | NM_000528 | 2.20 | Mannosidase alpha class 2B member 1. |
| CARKL | NM_013276 | 2.20 | Carbohydrate kinase-like protein. |
| HSDL1 | NM_031463 | 2.20 | hydroxysteroid dehydrogenase like 1. |
| EDN1 | NM_001955 | 2.20 | Endothelin-1 precursor. |
| ELMO3 | NM_024712 | 2.19 | Engulfment and cell motility protein 3. |
| TNNC1 | NM_003280 | 2.19 | Troponin C, slow skeletal and cardiac muscles . |
| USP42 | XM_374396 | 2.19 | Ubiquitin-specific-processing protease 42. |
| UBXD1 | NM_025241 | 2.19 | UBX domain-containing protein 1. |
| AMY2A | NM_000699 | 2.19 | Amylase, alpha 2A (pancreatic). |
| SAMD14 | NM_174920 | 2.19 | Sterile alpha motif domain-containing protein 14. |
| SUV39H1 | NM_003173 | 2.18 | Suppressor of variegation 3-9 homolog 1. |
| FEM1A | NM_018708 | 2.18 | fem-1 homolog a. |
| E2F2 | NM_004091 | 2.18 | Transcription factor E2F2. |
| LPCAT1 | NM_024830 | 2.18 | Lysophosphatidylcholine acyltransferase like 2. |
| UBE2Z | NM_023079 | 2.17 | ubiquitin-conjugating enzyme E2Z. |
| VASP | NM_003370 | 2.17 | Vasodilator-stimulated phosphoprotein. |
| RAD54L | NM_003579 | 2.16 | DNA repair and recombination protein RAD54-like. |
| LRRC52 | NM_001005214 | 2.16 | Leucine-rich repeat-containing protein 52 precursor. |
| C20orf149 | NM_024299 | 2.16 | Chromosome 20 open reading frame 149. |
| PXN | NM_002859 | 2.16 | Paxillin. |
| ANKRD47 | NM_198471 | 2.15 | Ankyrin repeat domain-containing protein 47. |
| C1orf188 | NM_173795 | 2.15 | Chromosome 1 open reading frame 188. |
| PUSL1 | NM_153339 | 2.15 | tRNA pseudouridine synthase-like 1. |
| DULLARD | NM_015343 | 2.15 | dullard homolog. |
| SEC24C | NM_004922 | 2.15 | Protein transport protein Sec24C. |
| KIF21B | XM_371332 | 2.14 | Kinesin family member 21B. |
| TGM1 | NM_000359 | 2.14 | Transglutaminase-1. |
| FBXW9 | NM_032301 | 2.14 | F-box and WD-40 domain protein 9. |
| PARD6A | NM_016948 | 2.14 | Partitioning defective 6 homolog alpha. |
| SEC61A1 | NM_013336 | 2.13 | Protein transport protein Sec61 subunit alpha isoform 1. |
| CCDC74A | NM_138770 | 2.13 | coiled-coil domain containing 74A. |
| SUPV3L1 | NM_003171 | 2.13 | suppressor of var1, 3-like 1. |
| C1QTNF6 | NM_031910 | 2.13 | Complement C1q tumor necrosis factor-related protein 6 precursor. |
| HEXDC | NM_173620 | 2.13 | hexosaminidase (glycosyl hydrolase family 20, catalytic domain) containing. |
| ZNF236 | NM_007345 | 2.12 | Zinc finger protein 236. |
| SFN | NM_006142 | 2.12 | 14-3-3 protein sigma (Stratifin). |
| TBCD | NM_005993 | 2.11 | Tubulin-specific chaperone D. |
| ZNF35 | NM_003420 | 2.11 | Zinc finger protein 35. |
| RASSF4 | NM_032023 | 2.11 | Ras association domain-containing protein 4. |
| CHMP7 | NM_152272 | 2.11 | Protein CHMP7. |
| BAP1 | NM_004656 | 2.11 | BRCA1- associated protein 1. |
| GLRA4 | XM_497139 | 2.10 | Glycine receptor, alpha 4 subunit. |
| NP_001027561.1 | NM_001004351 | 2.10 | Similar to Williams Beuren syndrome chromosome region 19. |
| C9orf75 | NM_173691 | 2.10 | Chromosome 9 open reading frame 75 |
| NP_001025056.1 | XM_496335 | 2.10 | MGC10334 protein. |
| LEPREL1 | NM_018192 | 2.10 | Prolyl 3-hydroxylase 2 precursor (Leprecan-like protein 1). |
| TAF3 | XM_291729 | 2.09 | Transcription initiation factor TFIID subunit 3 (TBP-associated factor 3). |
| FICD | NM_007076 | 2.09 | Huntingtin interacting protein E (FIC domain containing). |
| TRUB2 | NM_015679 | 2.09 | TryB pseudouridine (psi) synthase homolog 2. |
| DNAJC18 | NM_152686 | 2.09 | DnaJ homolog subfamily C member 18. |
| ZNF500 | NM_021646 | 2.09 | Zinc finger protein 500. |
| COG4 | NM_015386 | 2.09 | Conserved oligomeric Golgi complex component 4. |
| C18orf4 | NM_032160 | 2.08 | Chromosome 18 open reading frame 4. |
| SURF2 | NM_017503 | 2.08 | Surfeit locus protein 2. |
| EHD1 | NM_006795 | 2.08 | EH domain-containing protein 1. |
| HTRA3 | NM_053044 | 2.08 | Probable serine protease HTRA3 precursor (HtrA serine peptidase 3). |
| PTPRQ | NM_001145026 | 2.08 | Protein tyrosine phosphatase RQ (Fragment). |
| APOM | NM_019101 | 2.08 | Apolipoprotein M. |
| CASC3 | NM_007359 | 2.08 | Cancer susceptibility candidate gene 3 protein. |
| ANKS3 | NM_133450 | 2.07 | Ankyrin repeat and SAM domain-containing protein 3. |
| PRMT7 | NM_019023 | 2.07 | Protein arginine N-methyltransferase 7. |
| PHKG2 | NM_000294 | 2.07 | Phosphorylase kinase subunit gamma 2. |
| FKBP4 | NM_002014 | 2.06 | FK506-binding protein 4. |
| PLCXD1 | NM_018390 | 2.06 | phosphatidylinositol-specific phospholipase C, X domain containing 1. |
| BAZ2B | NM_013450 | 2.06 | Bromodomain adjacent to zinc finger domain protein 2B. |
| DHX37 | NM_032656 | 2.06 | DEAH box protein 37. |
| ZNF613 | NM_024840 | 2.06 | Zinc finger protein 613. |
| KRTAP10-10 | NM_181688 | 2.06 | Keratin-associated protein 10-10. |
| TMEM19 | NM_018279 | 2.05 | transmembrane protein 19. |
| PNPO | NM_018129 | 2.05 | Pyridoxine-5'-phosphate oxidase. |
| SURF4 | NM_033161 | 2.05 | Surfeit locus protein 4. |
| RFWD2 | NM_022457 | 2.05 | RING finger and WD repeat domain protein 2. |
| AHCY | NM_000687 | 2.05 | Adenosylhomocysteinase. |
| C9orf140 | NM_178448 | 2.05 | Chromosome 9 open reading frame 140. |
| HS1BP3 | NM_022460 | 2.05 | HCLS1 binding protein 3. |
| ZNF766 | NM_001010851 | 2.05 | zinc finger protein 766. |
| PAQR4 | NM_152341 | 2.04 | Progestin and adipoQ receptor family member 4. |
| FBLN1 | NM_006486 | 2.04 | Fibulin-1 precursor. |
| SERTAD1 | NM_013376 | 2.04 | SERTA domain-containing protein 1. |
| PPM1F | NM_014634 | 2.04 | Protein phosphatase 1F. |
| NP_001008396.1 | NM_001008395 | 2.04 | similar to CG14977-PA (LOC389541). |
| ANXA6 | NM_001155 | 2.04 | Annexin A6. |
| LMO6 | NM_006150 | 2.04 | LIM domain only protein 6. |
| RECQL4 | NM_004260 | 2.04 | RecQ protein-like 4. |
| DHCR24 | NM_014762 | 2.04 | 24-dehydrocholesterol reductase precursor. |
| LMAN2L | NM_030805 | 2.03 | Lectin, mannose-binding 2-like. |
| KRT16 | NM_005557 | 2.03 | Keratin, type I cytoskeletal 16. |
| UNC93B1 | NM_030930 | 2.03 | UNC93 homolog B1. |
| ZFYVE19 | NM_032850 | 2.03 | Zinc finger FYVE domain-containing protein 19. |
| CAMKK2 | NM_006549 | 2.03 | Calcium/calmodulin-dependent protein kinase kinase 2. |
| APOC1 | NM_001645 | 2.03 | Apolipoprotein C-I precursor. |
| DAOA | NM_172370 | 2.03 | D-amino acid oxidase activator. |
| EDC3 | NM_025083 | 2.02 | Enhancer of mRNA decapping protein 3. |
| WDR32 | NM_024345 | 2.02 | WD repeat domain 32. |
| KRT12 | NM_000223 | 2.02 | Keratin, type I cytoskeletal 12. |
| ACOT8 | NM_005469 | 2.02 | Acyl-coenzyme A thioesterase 8. |
| TRIM45 | NM_025188 | 2.02 | Tripartite motif-containing protein 45. |
| JOSD1 | NM_014876 | 2.02 | Josephin domain-containing 1. |
| TMEM71 | NM_144649 | 2.01 | transmembrane protein 71. |
| RNF24 | NM_007219 | 2.01 | RING finger protein 24. |
| ZFAND2B | NM_138802 | 2.01 | zinc finger, AN1-type domain 2B. |
| USP40 | NM_018218 | 2.01 | Ubiquitin-specific-processing protease 40. |
| SYDE1 | NM_033025 | 2.01 | synapse defective 1, Rho GTPase, homolog 1. |
| CLSTN1 | NM_001009566 | 2.01 | Calsyntenin-1 precursor. |
| MC1R | NM_002386 | 2.01 | Melanocortin 1 receptor precursor. |
| FCGR2A | NM_021642 | 2.01 | Low affinity immunoglobulin gamma Fc region receptor II-a precursor. |
| PHGDH | NM_006623 | 2.00 | D-3-phosphoglycerate dehydrogenase. |
| VPS18 | NM_020857 | 2.00 | Vacuolar protein sorting-associated protein 18. |
| Q6ZPC6_HUMAN | NM_201401 | 2.00 | CDNA FLJ26056 fis, clone PRS03239. |
| H1FX | NM_006026 | 2.00 | Histone H1x. |
