# Supplementary material for: Circadian Disruption Accelerates Tumor Growth and Angio/Stromagenesis through a Wnt Signaling Pathway
Source: PLoS One. 2010 Dec 23;5(12):e15330. doi: 10.1371/journal.pone.0015330 (PMC3009728; doi:10.1371/journal.pone.0015330)
Supplement: Table S2 — Primers used for construction of reporter plasmid, protein expression plasmid and semi-quantitative RT-PCR. (DOC) [file pone.0015330.s006.doc]

| Table S2. Primers used for constraction of reporter plasmid, protein expression plasmid and semi-quantitative RT-PCR. | | | | |
| --- | --- | --- | --- | --- |
|  |  |  |  |  |
| Name | Gene accession |  | Primer sequence | Product size, bp |
| h-WNT10A  (promoter cloning) |  | Fw | 5'-AGATCTCAGCGAGAAGTAGACCTGAGCCAG-3' | 1256 |
|  |  | Rv | 5'-AAGCTTGGGGCAGCACCCCCGGGCACCGCC-3' |  |
| h-WNT10A  (cDNA cloning) | NM_025216 | Fw | 5'-GGATCCATGGGCAGCGCCCACCCTCGCCCC-3' | 1254 |
|  |  | Rv | 5'-TCACTTACACACATTCACCCACTCTGTAAC-3' |  |
| h-WNT10A  (RT-PCR first) | NM_025216 | Fw | 5'-GAGGCCTTCCGTAGGAAGCTGCACCGC-3' | 573 |
|  |  | Rv | 5'-CACGGTGCCCGCCGAGTCCAGGCGCGG-3' |  |
| h-WNT10A  (RT-PCR second) | NM_025216 | Fw | 5'-GTCCCGGAACACCCAGCCCTGCCCA-3' | 437 |
|  |  | Rv | 5'-ACCAGGTCGGCGGGGCTGGCCCGT-3' |  |
| m-Wnt10a | NM_009518 | Fw | 5'-GGTCCCTGAACACCCGGCCATACTTC-3' | 438 |
|  |  | Rv | 5'-ACCAGGTCGGAGTGGCTGGCCCTG-3' |  |
| ANGPL4 | NM_139314 | Fw | 5'-ACAGCCGCCTGGCCGTGCAGC-3' | 428 |
|  |  | Rv | 5'-GGCCCAGCCAGGACGCTAGGAGGC-3' |  |
| RB1 | NM_000321 | Fw | 5'-AGTGCTGAAGGAAGCAACCCTCCTAAACC-3' | 546 |
|  |  | Rv | 5'-AAGTAGTCAGACAGGCCTGGGTTATCAGG-3' |  |
| VEGFA | NM_001025366 | Fw | 5'-AAACACAGACTCGCGTTGCAAGGCG-3' | 335 |
|  |  | Rv | 5'-TCCAATTCCAAGAGGGACCGTGCTG-3' |  |
| VEGFB | NM_003377 | Fw | 5'-CTTAGAGCTCAACCCAGACACCTGC-3' | 294 |
|  |  | Rv | 5'-TGAGCTGGTATGTGACCCCTCTTGC-3' |  |
| YB-1 | NM_004559 | Fw | 5'-ACAAGAAGGTCATCGCAACGAAGG-3' | 236 |
|  |  | Rv | 5'-ACCAGGACCTGTAACATTTGCTGC-3' |  |
| h-β-actin | NM_001101 | Fw | 5'-GAGATGGCCACGGCTGCTTCCAGC-3' | 299 |
|  |  | Rv | 5'-ATTGTGCTGGGTGCCAGGGCAGTG-3' |  |
| m-β-actin | NM_007393 | Fw | 5'-GAGATGGCCACTGCCGCATCCTCT-3' | 299 |
|  |  | Rv | 3'-ATGGTGCTAGGAGCCAGAGCAGTA-3' |  |
| h-: human; m-: mouse | | |  |  |
