# Supplementary material for: Circadian Disruption Accelerates Tumor Growth and Angio/Stromagenesis through a Wnt Signaling Pathway
Source: PLoS One. 2010 Dec 23;5(12):e15330. doi: 10.1371/journal.pone.0015330 (PMC3009728; doi:10.1371/journal.pone.0015330)
Supplement: Table S3 — Double-stranded RNA 25-base pair oligonucleotides used for WNT10A kockdown analysis. (DOC) [file pone.0015330.s007.doc]

| Table S3. Double-stranded RNA 25-base pair oligonucleotides used for WNT10A kockdown analysis. | | |
| --- | --- | --- |
|  |  |  |
|  |  |  |
| Name |  | Sequence |
| WNT10A siRNA #1 | sense | 5'-UGAAGCCUCAUUCUCGCGUGGAUGU-3' |
|  | antisense | 5'-ACAUCCACGCGAGAAUGAGGCUUCA-3' |
| WNT10A siRNA #2 | sense | 5'-UAGGCACACUGUGUUGGCAUUGAGC-3' |
|  | antisense | 5'-GCUCAAUGCCAACACAGUGUGCCUA-3' |
